# Supplementary material for: The distinction of CPR bacteria from other bacteria based on protein family content
Source: Nat Commun. 2019 Sep 13;10:4173. doi: 10.1038/s41467-019-12171-z (PMC6744442; doi:10.1038/s41467-019-12171-z)
Supplement: Supplementary file 1 — Supplementary Information [file 41467_2019_12171_MOESM1_ESM.pdf]

Supplementary Information

The distinction of CPR bacteria from other bacteria based on protein family content

Méheust *et al*.

This PDF file includes:
Supplementary Figures 1-15
Supplementary Table 1
Supplementary References

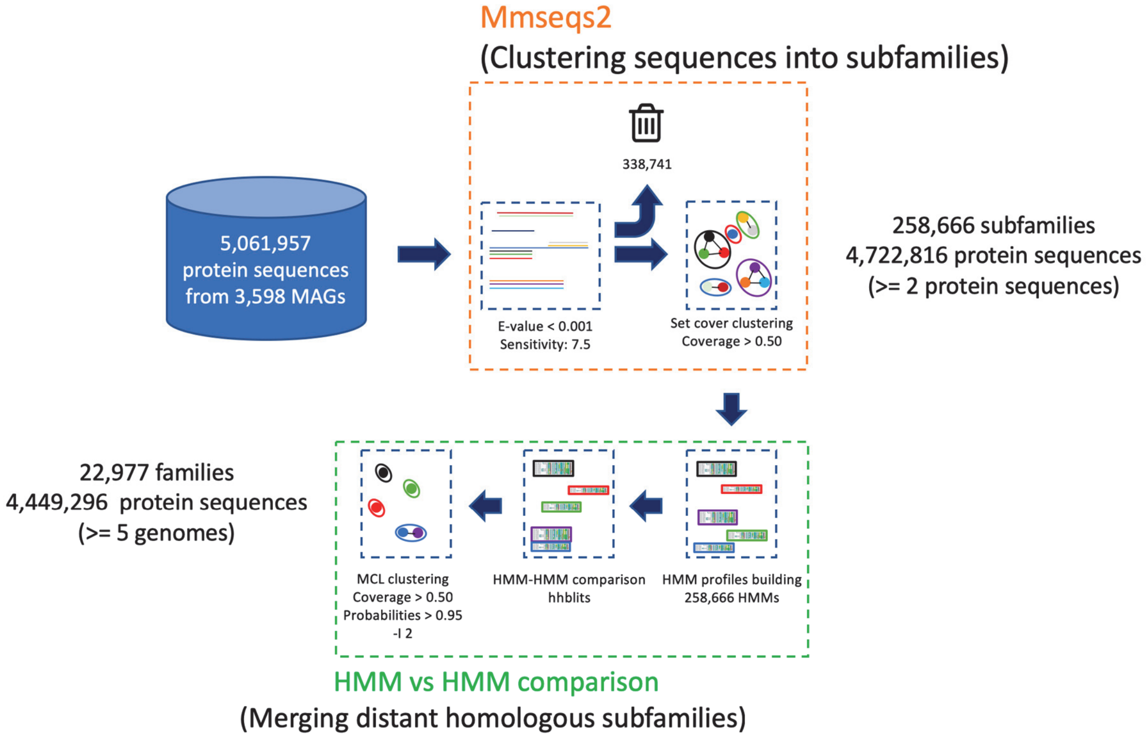

Supplementary Figure 1. The protein clustering pipeline used in the study. MAGs: metagenome-assembled genomes.

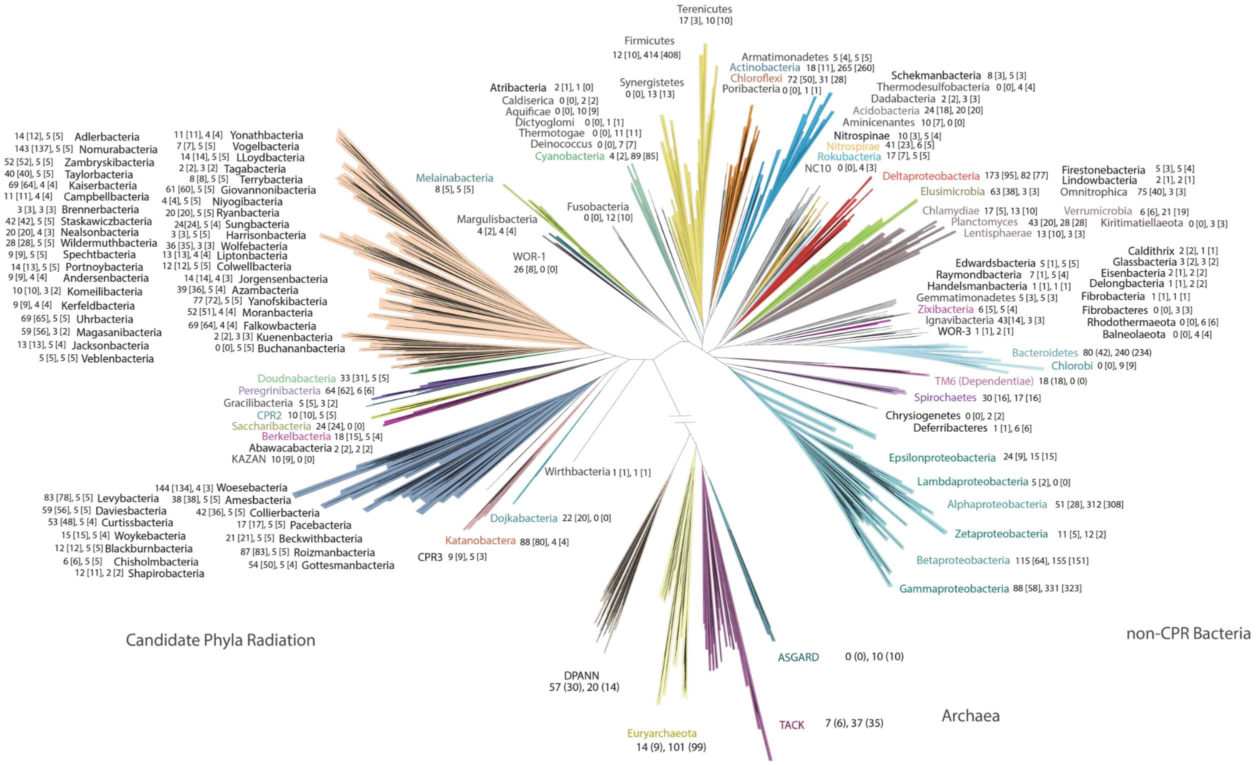

Supplementary Figure 2. Tree illustrating phylogenetic sampling used in this study (the diagram is based on a tree published recently in ref. [1]). For each phylum, the number of genomes and near-complete genomes (square brackets) is reported for the two datasets.

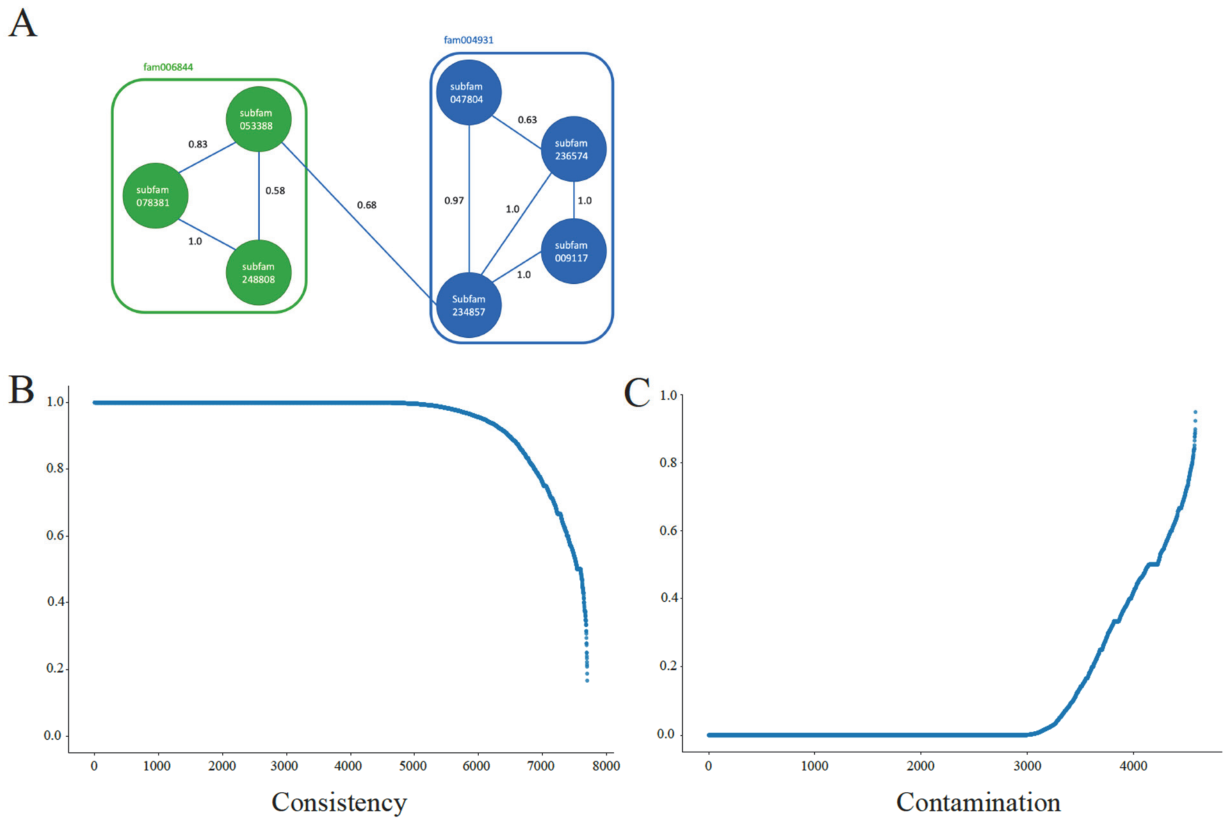

Supplementary Figure 3. Quality assessment of the protein clustering. A. Subfamilies network used for the MCL clustering and that defined the two RPL2 families. B. Consistency between the KEGG annotations and the protein families. For each of the 7700 annotations, we reported the family which contains the highest percentage of protein members annotated with that KEGG annotation. Each dot represents a KEGG annotation, the y-axis represents the highest percentage. C. Contamination of the protein families. For each family with proteins having KEGG annotations, we computed the percentage of the proteins that have KEGG annotations different than the most abundant one, this percentage defined the annotation admixture (y-axis). Each dot represents a protein family.

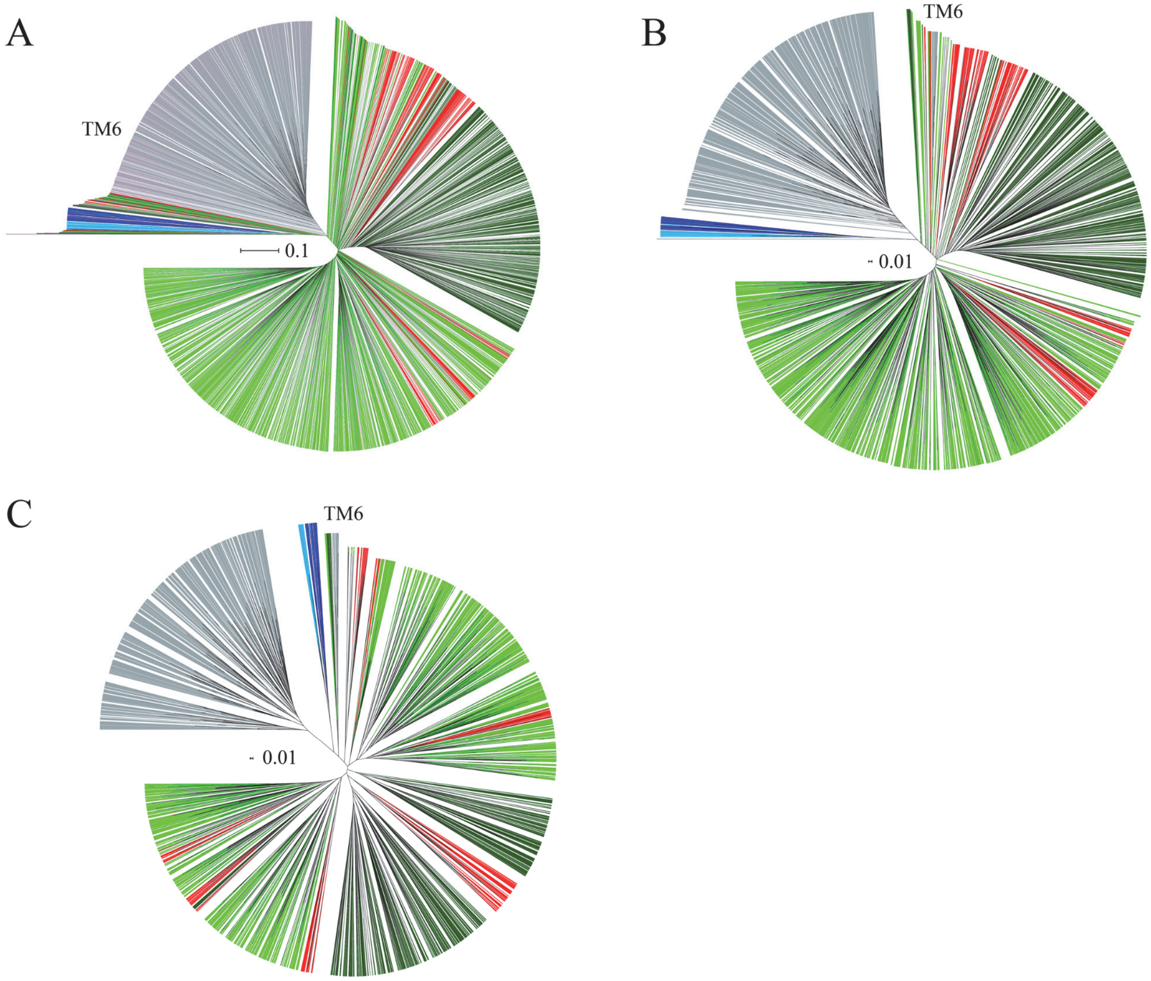

Supplementary Figure 4. Trees resulting from the hierarchical clustering of the genomes based on the distributions of 22,977 protein families in 2890 near-complete and non-redundant genomes from Parcubacteria (green), Microgenomates (darkgreen), other CPR bacteria (red), non-CPR bacteria (grey), DPANN (blue) and non-DPANN archaea (lightblue). Three methods of agglomerative hierarchical clustering were showed. A. Single linkage. B. Average linkage. C. Complete linkage.

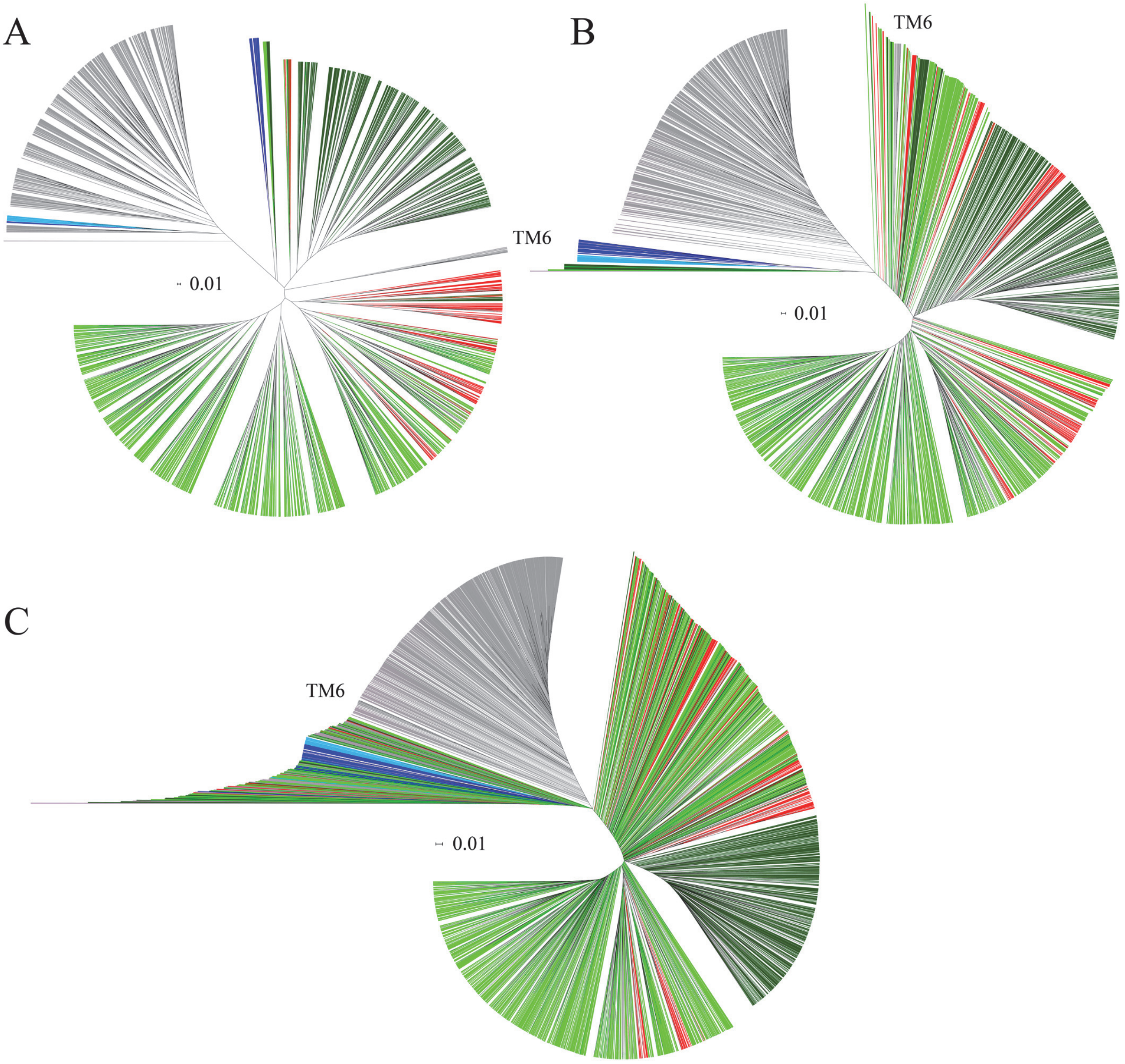

Supplementary Figure 5. Trees resulting from the hierarchical clustering of the genomes based on the distributions of 921 protein families in 2890 near-complete and non-redundant genomes from Parcubacteria (green), Microgenomates (darkgreen), other CPR bacteria (red), non-CPR bacteria (grey), and DPANN (blue) and non-DPANN archaea (lightblue). Three methods of agglomerative hierarchical clustering were showed. A. Complete linkage. B. Average linkage. C. Single linkage.

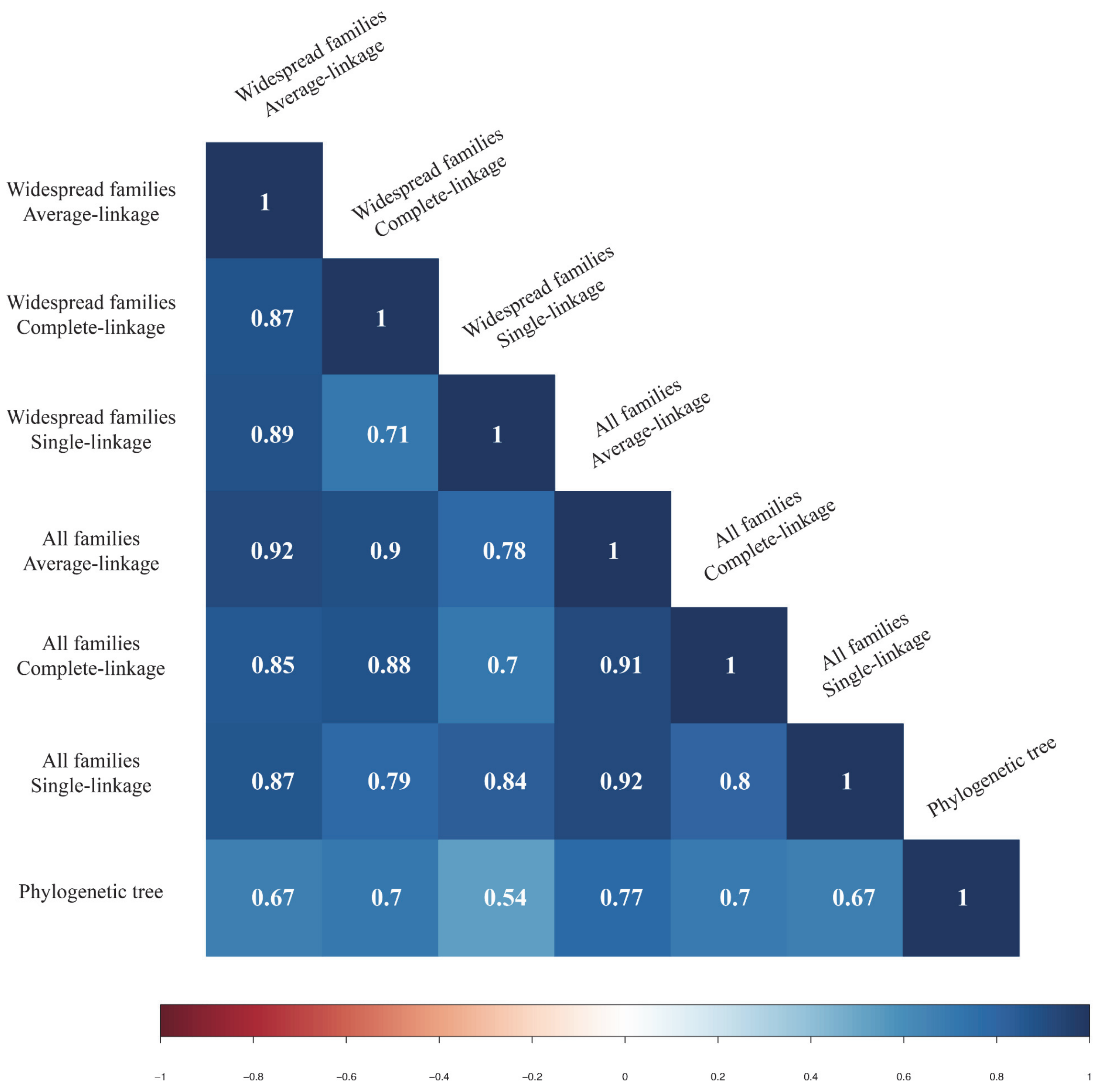

Supplementary Figure 6. Correlation plot of 6 trees obtained from 3 different hierarchical clustering methods (complete linkage, average linkage and single linkage) and two different families sets of the initial dataset (the 22,977 “all families” set and the 921 “widespread families” set). Maximun-likelihood tree based on RAxML is also showed (“Phylogenetic tree”). Correlations are based on cophenetic distance matrices between pairs of trees. Positive correlations are displayed in blue and negative correlations in red color. Color intensity is proportional to the correlation coefficient.

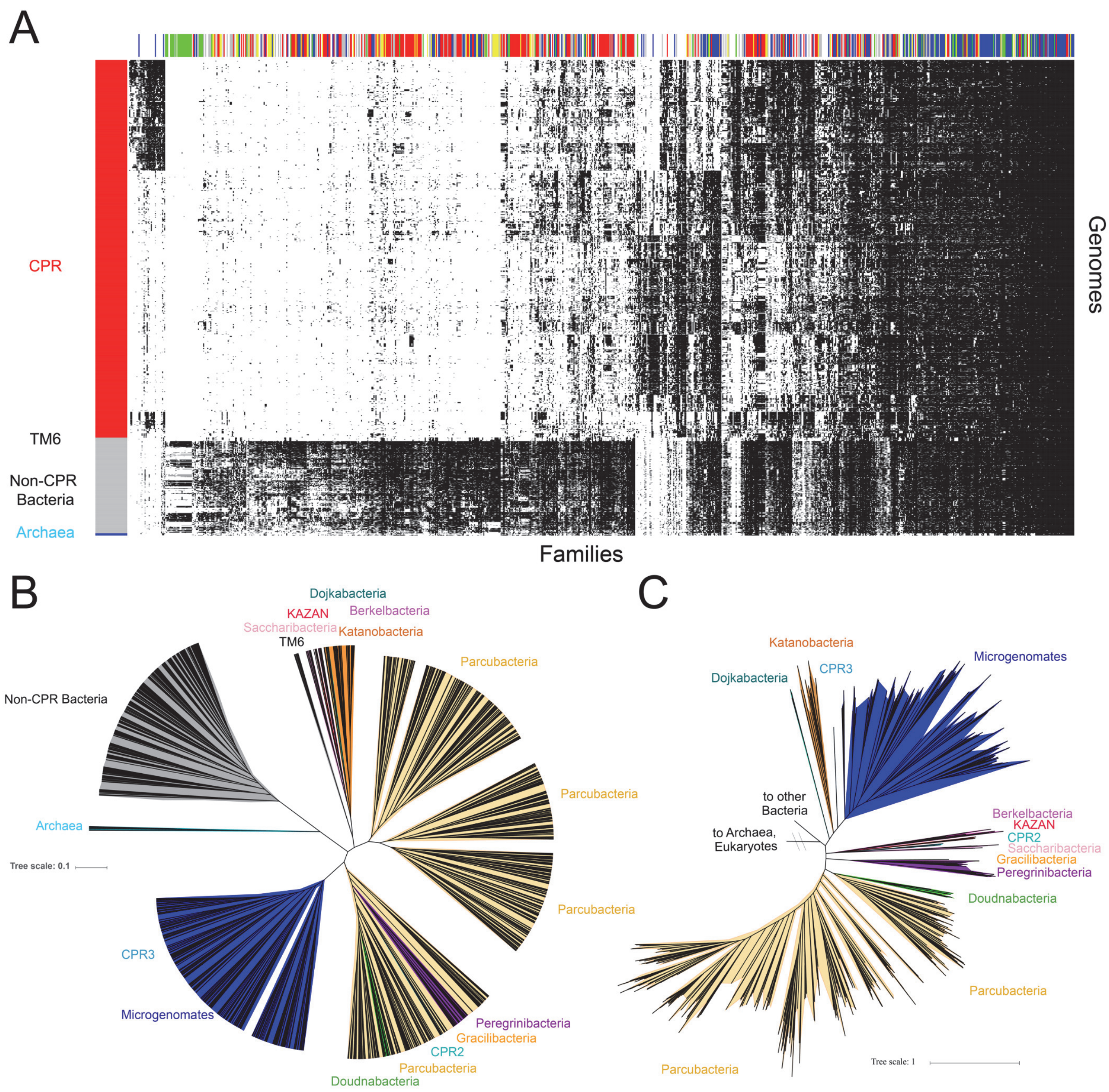

Supplementary Figure 7. The distribution of 921 widely distributed protein families across the 1,966 high-quality genomes. (A) The distribution of 921 widely distributed protein families (columns) in 1,966 genomes (rows) from CPR bacteria (red), non-CPR bacteria (gray), and a few archaea (light blue) in a reference set with extensive sampling of genomes from metagenomes (thus includes sequences from many candidate phyla) (completeness > 90%). Data are clustered based on the presence (black) / absence (white) profiles (Jaccard distance, complete linkage). Only draft-quality and non-redundant genomes were used. The colored top bar corresponds to the functional category of families (Metabolism: red, Genetic Information Processing: blue, Cellular

Processes: green, Environmental Information Processing: yellow, Organismal systems: orange, Unclassified: grey, Unknown: white). (B) Tree resulting from the hierarchical clustering of the genomes based on the distributions of proteins families in the panel A. (C) A phylogenetic tree of the CPR genomes present in the dataset. The maximum-likelihood tree was calculated based on the concatenation of 14 ribosomal proteins (L2, L3, L4, L5, L6, L14, L15, L18, L22, L24, S3, S8, S17, and S19) using the PROTCATLG model.

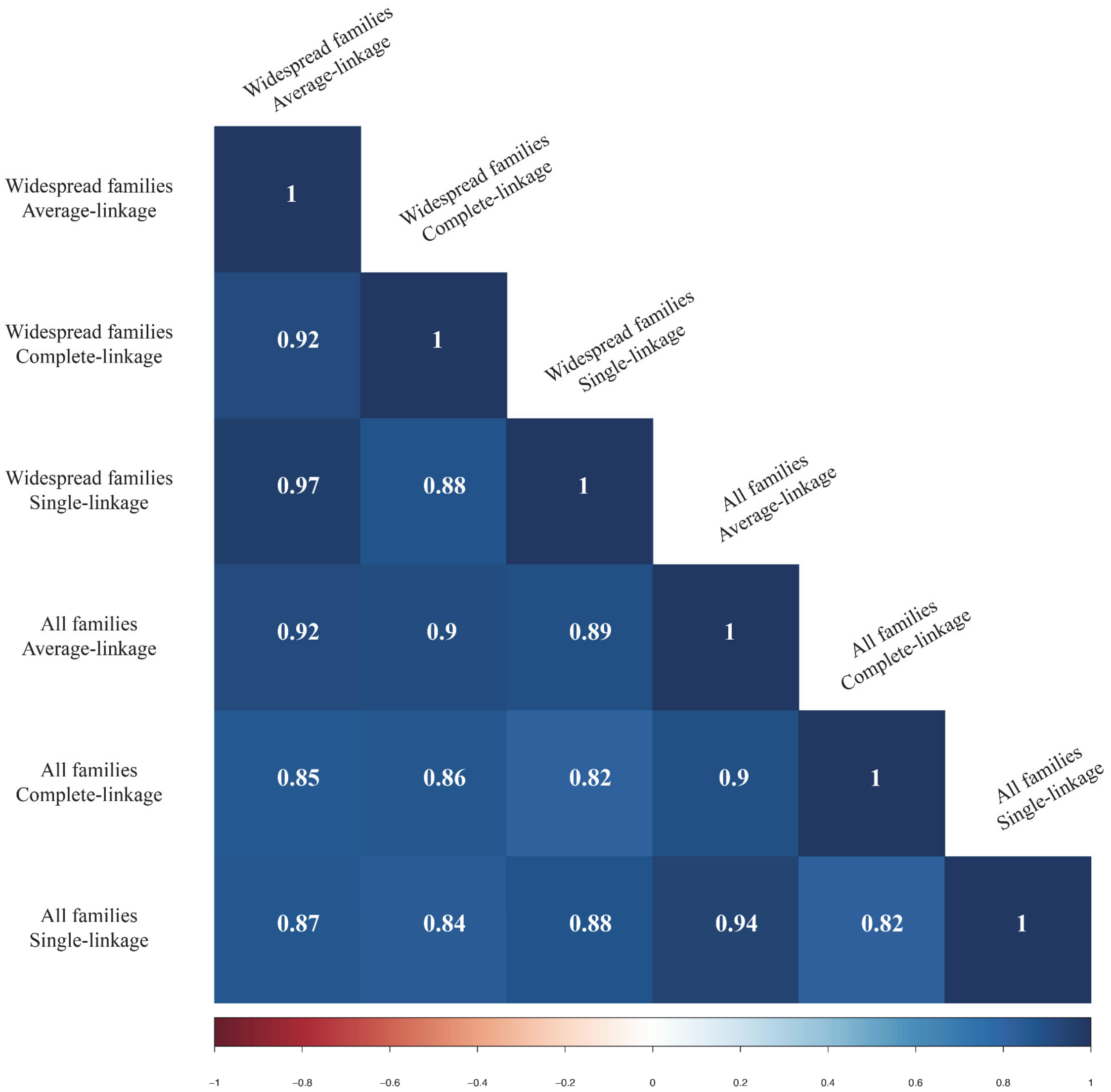

Supplementary Figure 8. Correlation plot of 6 trees obtained from 3 different hierarchical clustering methods (complete linkage, average linkage and single linkage) and two different families sets of the NCBI dataset (the 15,137 families set and the 921 widespread families set). Correlations are based on cophenetic distance matrices between pairs of trees. Positive correlations are displayed in blue and negative correlations in red color. Color intensity is proportional to the correlation coefficient.

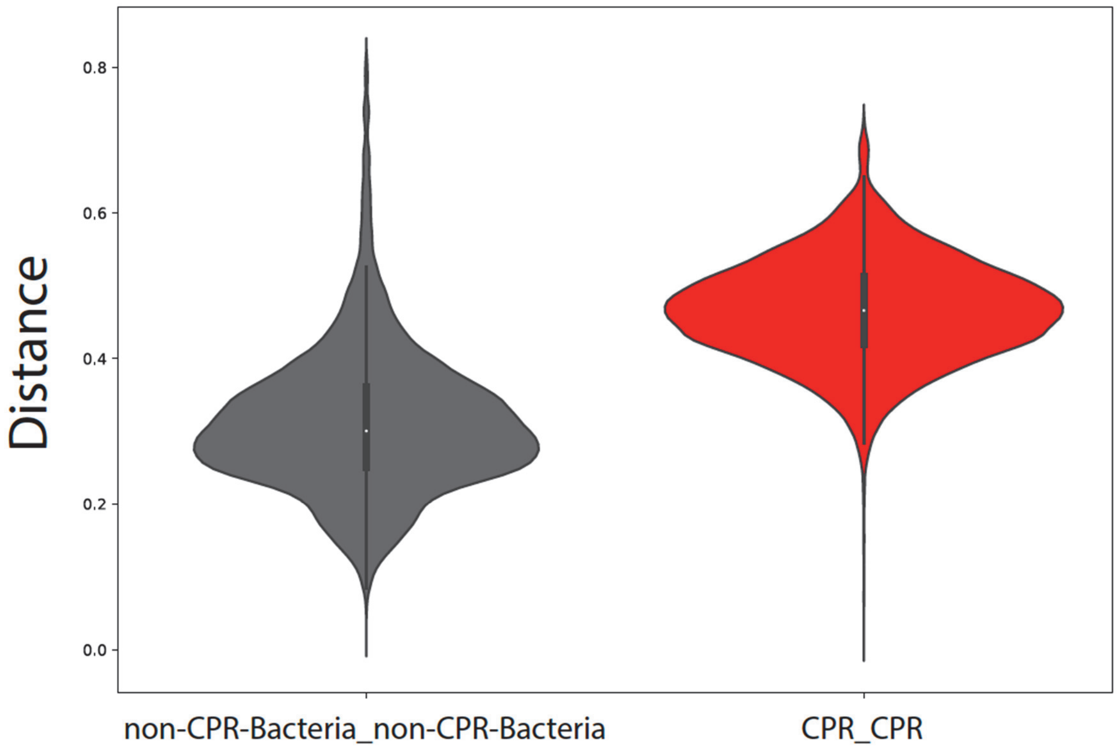

Supplementary Figure 9. Distributions of the Jaccard distances within the non-CPR-Bacteria (grey) and within the CPR. Jaccard distances have been calculated for each pair of CPR genomes (and non-CPR-Bacterial genomes) based on the presence / absence of the 921 families.

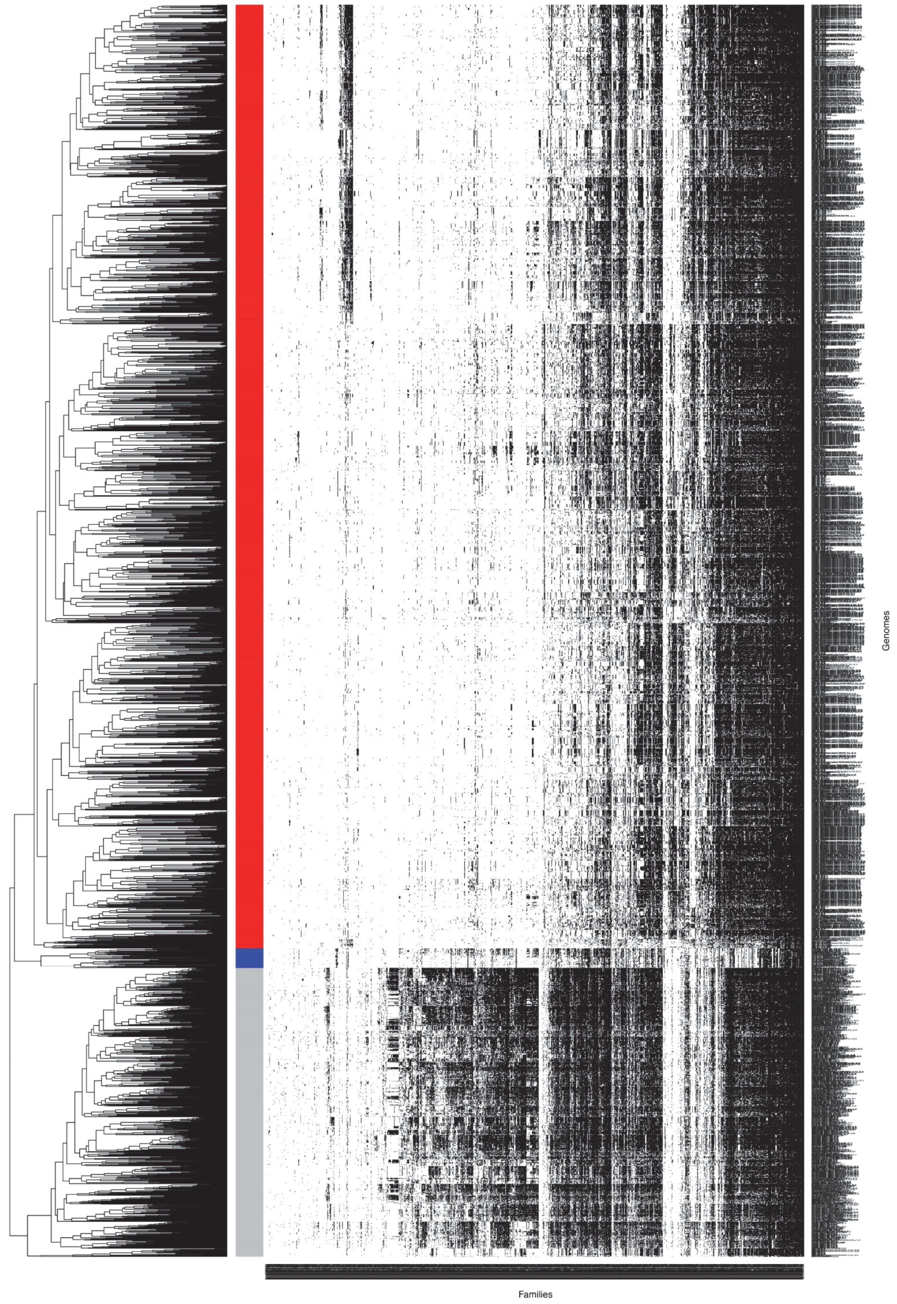

Supplementary Figure 10. The distribution of widely distributed 1216 protein clusters (columns) in 2890 genomes (rows) from CPR bacteria (red), non-CPR bacteria (grey), and a few archaea (blue) in a reference set with extensive sampling of genomes from metagenomes (thus includes sequences from many candidate phyla). Data are clustered based on the presence (black) / absence (white) profiles (Jaccard distance, complete linkage). Only near-complete and non-redundant genomes were showed. The non-CPR bacteria genomes (in grey) that are nested in the CPR (in red) correspond to the Dependentiae (TM6) genomes.

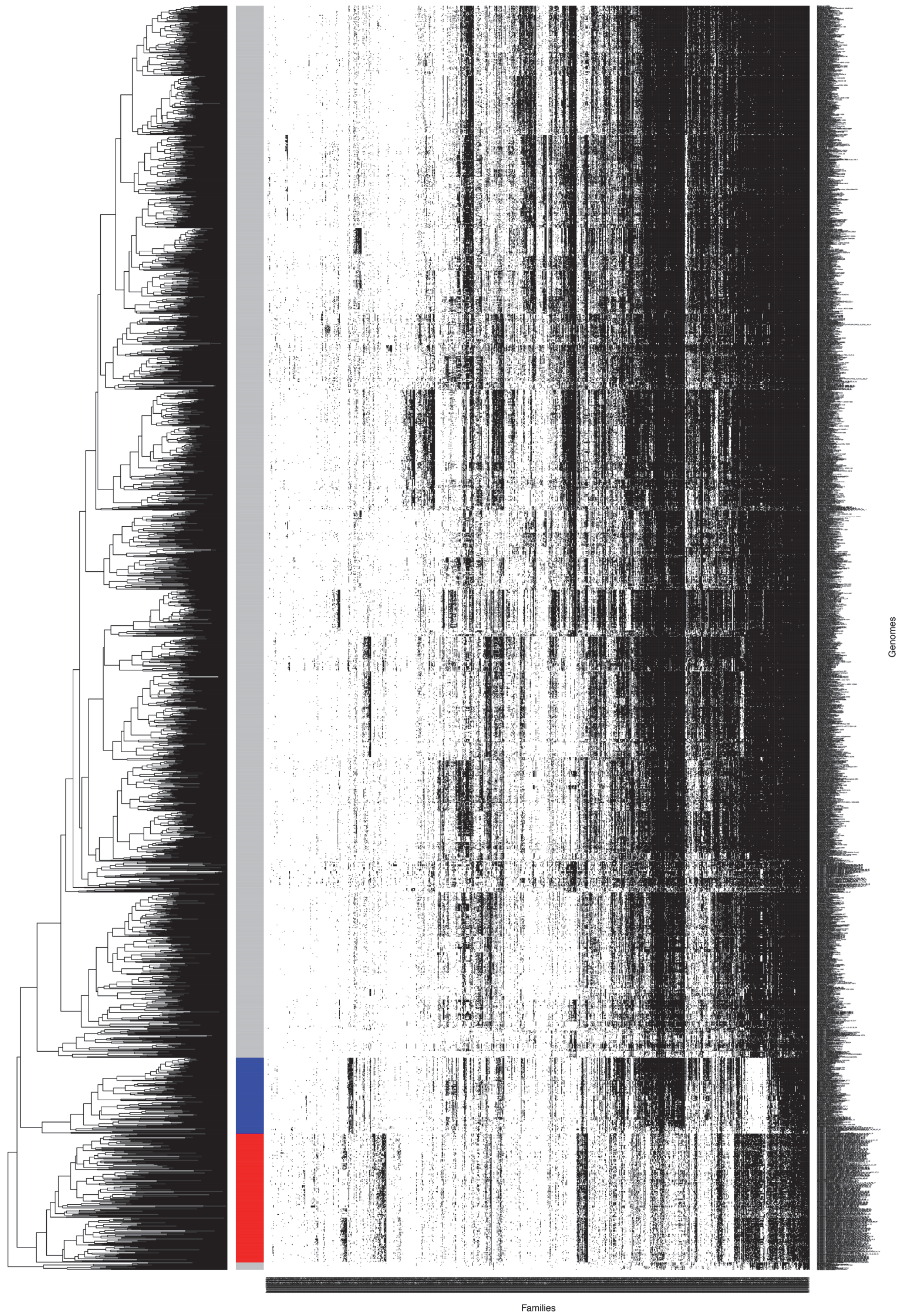

Supplementary Figure 11. The distribution of 1216 widely distributed protein clusters (columns) in 2,616 near-complete and non-redundant genomes (rows) from a reference set with extensive sampling of genomes from non-CPR bacteria (grey). Genomes are clustered based on the presence (black) / absence (white) profiles (Jaccard distance, complete linkage). CPR bacteria are colored in red, the Archaea are colored in blue.

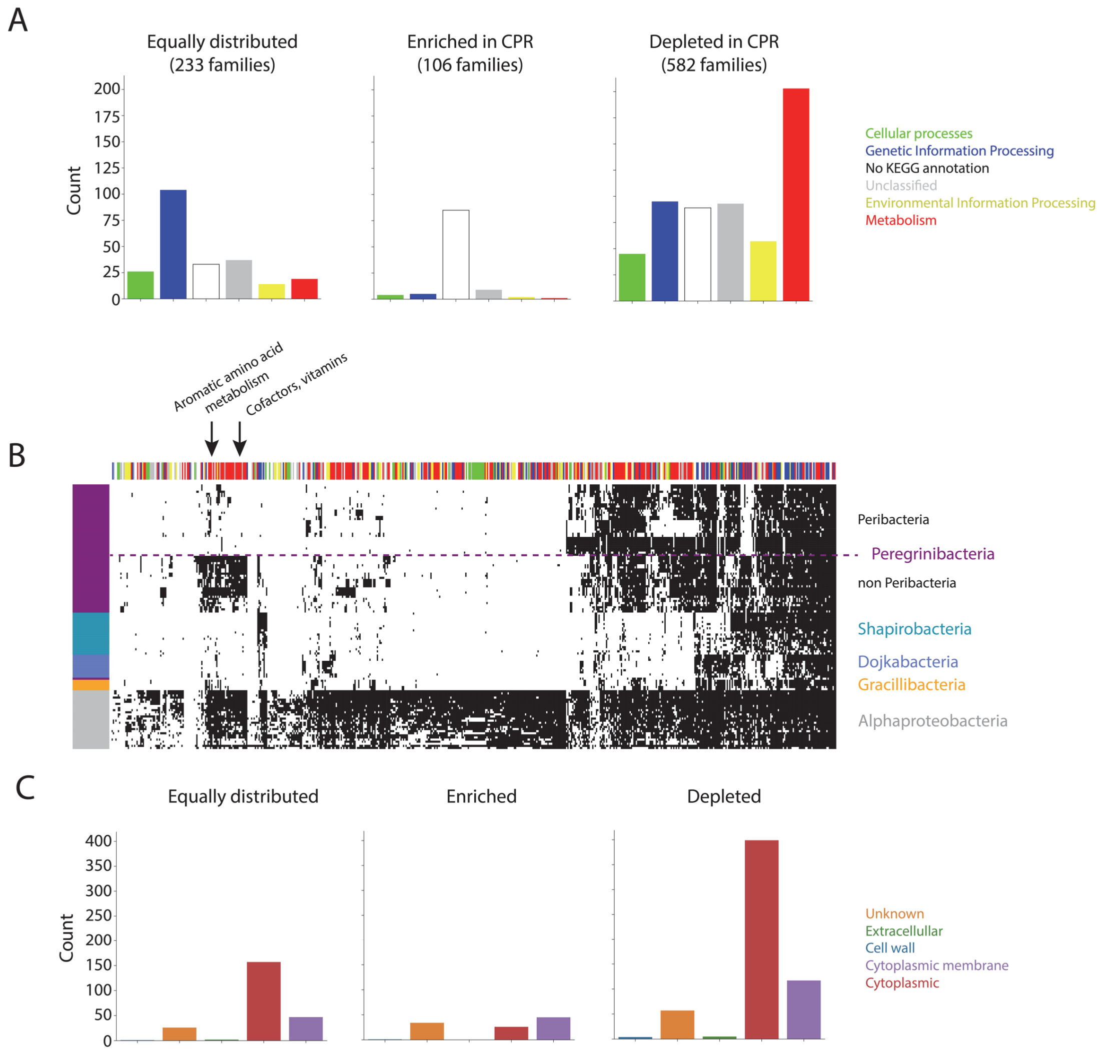

Supplementary Figure 12. (A) Barplots of the distributions of the functional categories of the 921 protein families. (B) Distribution of the 582 families that are depleted in CPR across 126 genomes from Peregrinibacteria (62), Shapirobacteria (11), Dojkabacteria (20), Gracilibacteria (5) and Alphaproteobacteria (28). The dashed line separates classes within the Peregrinibacteria. The order of the families and the genomes is the same as in Figure 3A. The colored top bar corresponds to the functional category of families (Metabolism: red, Genetic Information Processing: blue, Cellular Processes: green, Environmental Information Processing: yellow, Organismal systems: orange, Unclassified: grey, Unknown: white). (C) Barplots of the distributions of the cellular localizations of the 921 protein families.

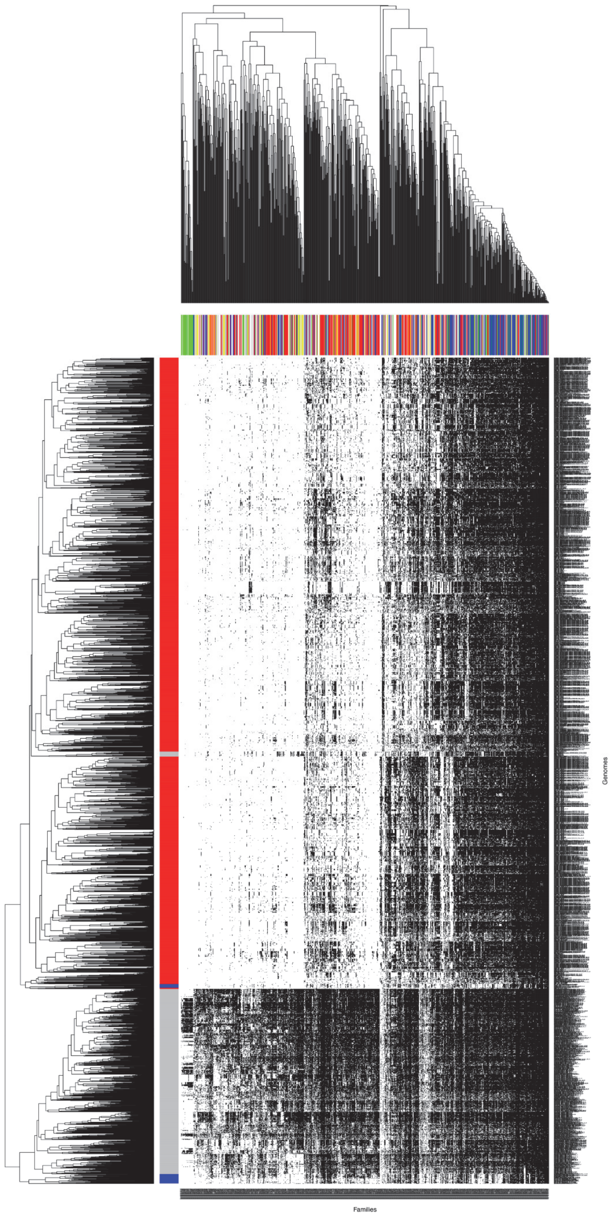

Supplementary Figure 13. The distribution of 815 widely distributed protein families (columns) in 2890 genomes (rows) from CPR bacteria (red), non-CPR bacteria (grey), and a few archaea (blue) in a reference set with extensive sampling of genomes from metagenomes (thus includes sequences from many candidate phyla). The 106 families that are enriched in CPR relative to non-CPR bacteria were not considered in this analysis. Genomes and families are clustered based on the presence (black) / absence (white) profiles (Jaccard distance, complete linkage). Only near-complete and non-redundant genomes were showed. The non-CPR bacteria genomes (in grey) that are nested in the CPR (in red) correspond to the Dependentiae (TM6) genomes.

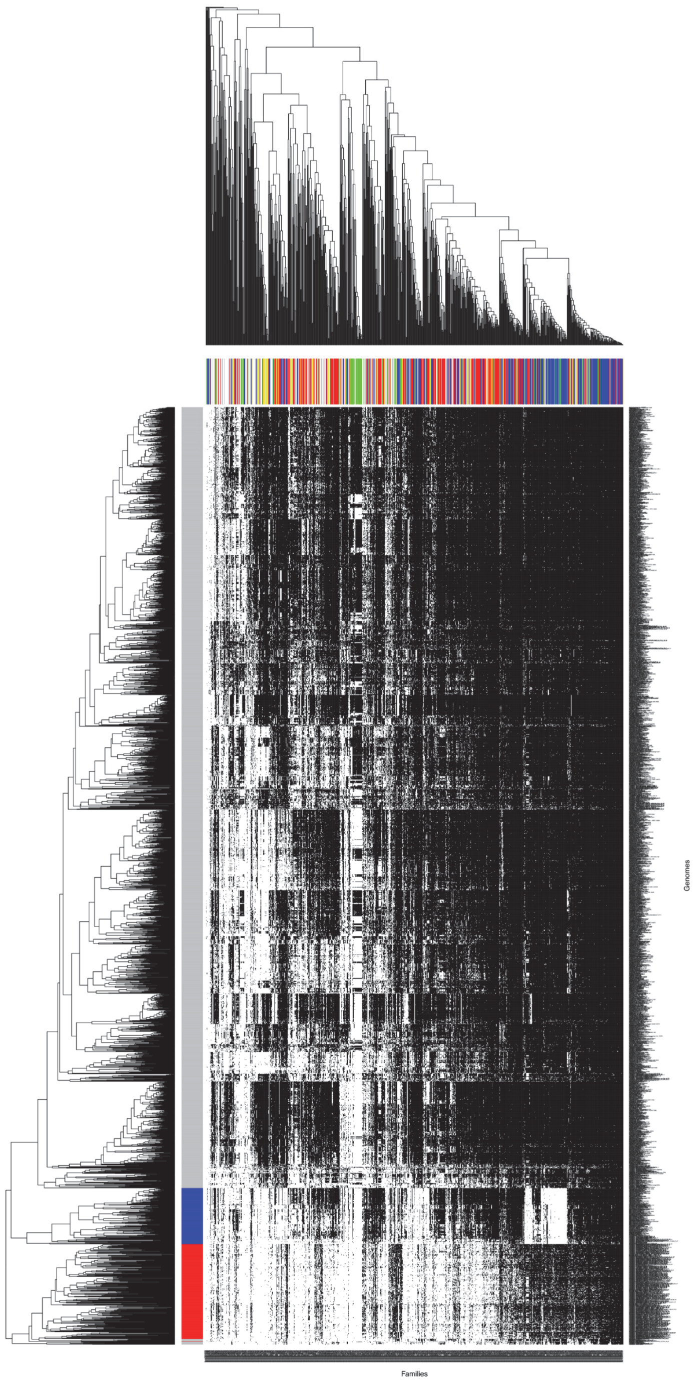

Supplementary Figure 14. The distribution of 815 widely distributed protein families (columns) in 2,616 genomes (rows) from CPR bacteria (red), non-CPR bacteria (grey), and archaea (blue) in a reference set from the NCBI genome database. The 106 families that are enriched in CPR relative to non-CPR bacteria were not considered in this analysis. Genomes and families are clustered based on the presence (black) / absence (white) profiles (Jaccard distance, complete linkage). Only near-complete and non-redundant genomes were showed.

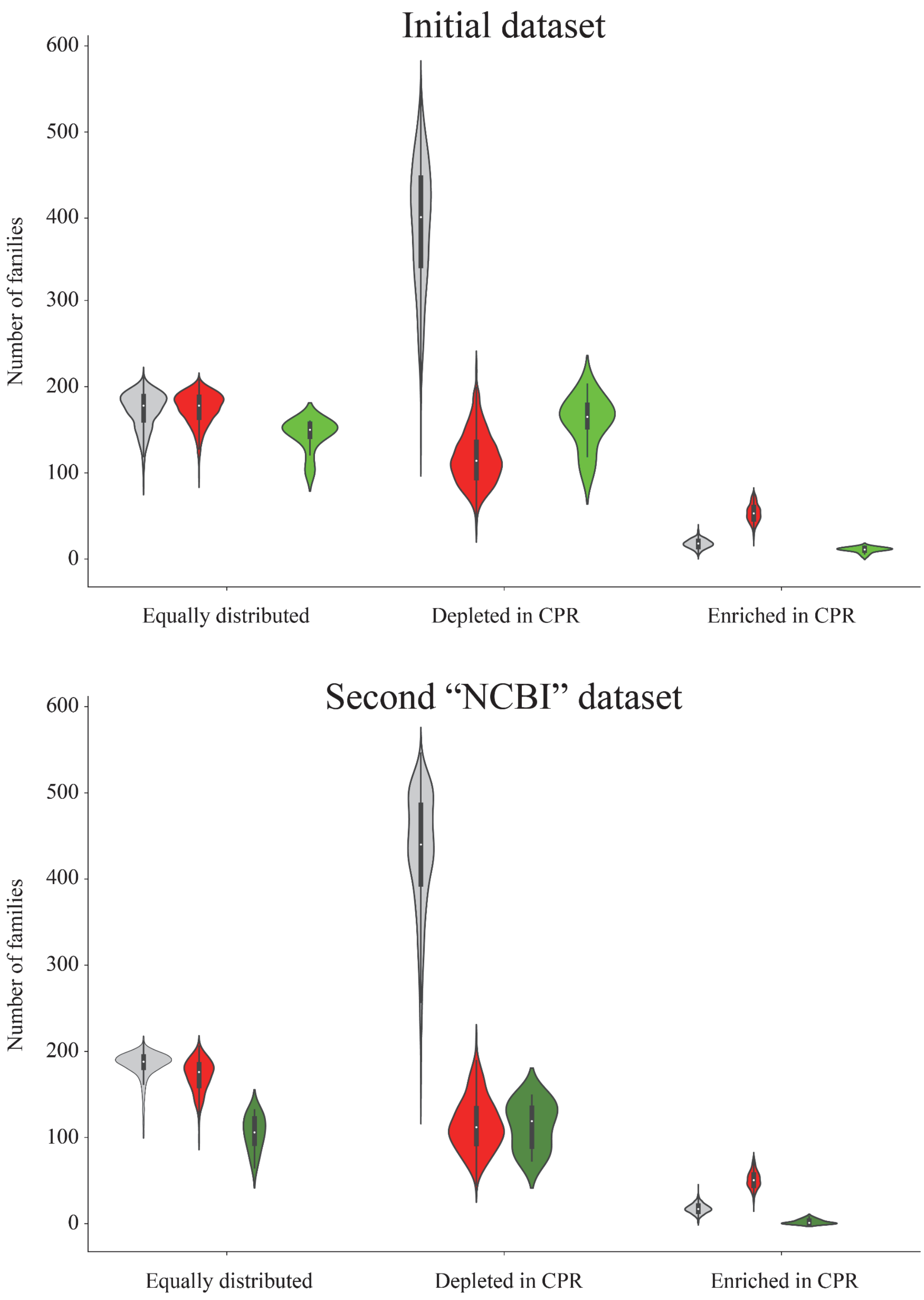

Supplementary Figure 15. Violin plots showing the number of families of three different sets of families (equally distributed, depleted and enriched sets) in CPR (red), non-CPR bacteria (grey), TM6 (green), Tenericutes plus highly-reduced genome (darkgreen).

Supplementary Table 1. Quality control of the protein clustering based on the 16 ribosomal proteins (RP). The rational is because those proteins are highly conserved, we expect to have each of them into a single cluster (i.e. one family per type of RP). This table summarized the results, each line corresponds to one RP protein. The proteins annotated as one of the 16 RP were retrieved using the corresponding Pfam accessions (PFAM accessions column). For 10 of them, all proteins cluster into one single family (# families column). The 6 remaining RP clusters in several families however there is always one family that contains the majority of the proteins (# proteins column).

RP	Pfam accessions	# families	# proteins	Family Accession	# unclustered proteins
RPL2	PF00181+PF03947	2	2673<br>837	fam004931<br>fam006844	1
RPL3	PF00297	1	3312	fam001238	2
RPL4	PF00573	1	3453	fam000859	5
RPL5	PF00281+PF00673	1	3522	fam001837	2
RPL6	PF00347+PF00347	1	3505	fam002354	1
RPL14	PF00238	1	3526	fam003363	0
RPL15	PF00828	1	3196	fam001057	1
RPL16	PF00252	1	3519	fam002413	1
RPL18	PF00861	1	3397	fam002211	2
RPL22	PF00237	1	3488	fam001242	3
RPL24	PF17136	1	3291	fam002119	2
RPS3	PF07650+PF00189	1	3499	fam002071	1
RPS8	PF00410	1	3489	fam002125	0
RPS10	PF00338	1	3271	fam002082	0
RPS17	PF00366	1	3510	fam001456	1
RPS19	PF00203	1	3461	fam002135	0

Supplementary References

1. Castelle, C. J. & Banfield, J. F. Major New Microbial Groups Expand Diversity and Alter our Understanding of the Tree of Life. *Cell* **172**, 1181–1197 (2018).
