# Supplementary material for: The distinction of CPR bacteria from other bacteria based on protein family content
Source: Nat Commun. 2019 Sep 13;10:4173. doi: 10.1038/s41467-019-12171-z (PMC6744442; doi:10.1038/s41467-019-12171-z)
Supplement: Supplementary file 3 — Reporting Summary [file 41467_2019_12171_MOESM3_ESM.pdf]

nature research

Corresponding author(s): Jill Banfield

Last updated by author(s): 2019/08/16

Reporting Summary

Nature Research wishes to improve the reproducibility of the work that we publish. This form provides structure for consistency and transparency in reporting. For further information on Nature Research policies, see Authors & Referees and the Editorial Policy Checklist.

Statistics

For all statistical analyses, confirm that the following items are present in the figure legend, table legend, main text, or Methods section.

n/a	Confirmed	
☐	☒	The exact sample size (*n*) for each experimental group/condition, given as a discrete number and unit of measurement
☐	☒	A statement on whether measurements were taken from distinct samples or whether the same sample was measured repeatedly
☐	☒	The statistical test(s) used AND whether they are one- or two-sided<br>*Only common tests should be described solely by name; describe more complex techniques in the Methods section.*
☒	☐	A description of all covariates tested
☐	☒	A description of any assumptions or corrections, such as tests of normality and adjustment for multiple comparisons
☐	☒	A full description of the statistical parameters including central tendency (e.g. means) or other basic estimates (e.g. regression coefficient) AND variation (e.g. standard deviation) or associated estimates of uncertainty (e.g. confidence intervals)
☐	☒	For null hypothesis testing, the test statistic (e.g. *F*, *t*, *r*) with confidence intervals, effect sizes, degrees of freedom and *P* value noted<br>*Give P values as exact values whenever suitable.*
☒	☐	For Bayesian analysis, information on the choice of priors and Markov chain Monte Carlo settings
☒	☐	For hierarchical and complex designs, identification of the appropriate level for tests and full reporting of outcomes
☒	☐	Estimates of effect sizes (e.g. Cohen's *d*, Pearson's *r*), indicating how they were calculated

Our web collection on statistics for biologists contains articles on many of the points above.

Software and code

Policy information about availability of computer code

Data collection	The present analysis is a meta-analysis. All genomes were already publicly available.
Data analysis	We used open-source and published programs. We used custom scripts to parse and filter the data. A script was written to run the protein clustering and has been made available on GitHub.

For manuscripts utilizing custom algorithms or software that are central to the research but not yet described in published literature, software must be made available to editors/reviewers. We strongly encourage code deposition in a community repository (e.g. GitHub). See the Nature Research guidelines for submitting code & software for further information.

Data

Policy information about availability of data

All manuscripts must include a data availability statement. This statement should provide the following information, where applicable:

- Accession codes, unique identifiers, or web links for publicly available datasets
- A list of figures that have associated raw data
- A description of any restrictions on data availability

All genomes used in the present study were publicly available

Field-specific reporting

Please select the one below that is the best fit for your research. If you are not sure, read the appropriate sections before making your selection.

☐ Life sciences ☐ Behavioural & social sciences ☒ Ecological, evolutionary & environmental sciences

Ecological, evolutionary & environmental sciences study design

All studies must disclose on these points even when the disclosure is negative.

Study description	The present analysis is a meta-analysis. All the data have been described in previous papers (see Methods).
Research sample	The present analysis is a meta-analysis. All the data have been described in previous papers (see Methods).
Sampling strategy	The present analysis is a meta-analysis. All the data have been described in previous papers (see Methods).
Data collection	Genomes were dereplicated and the quality of the genome assemblies was assessed (see Methods)
Timing and spatial scale	The present analysis is a meta-analysis. All the data have been described in previous papers.
Data exclusions	Only genomes with completeness > 70% and contamination < 10% were considered in the present study (see Methods).
Reproducibility	We used two different datasets, as well as several sets of parameters to run the protein clustering, to verify the reproducibility.
Randomization	Not relevant in the present study.
Blinding	One genome per taxonomic genus was randomly collected on the NCBI genome database.

Did the study involve field work? ☐ Yes ☒ No

Reporting for specific materials, systems and methods

We require information from authors about some types of materials, experimental systems and methods used in many studies. Here, indicate whether each material, system or method listed is relevant to your study. If you are not sure if a list item applies to your research, read the appropriate section before selecting a response.

Materials & experimental systems

n/a	Involved in the study
☒	☐ Antibodies
☒	☐ Eukaryotic cell lines
☒	☐ Palaeontology
☒	☐ Animals and other organisms
☒	☐ Human research participants
☒	☐ Clinical data

Methods

n/a	Involved in the study
☒	☐ ChIP-seq
☒	☐ Flow cytometry
☒	☐ MRI-based neuroimaging
