# Supplementary material for: The distinction of CPR bacteria from other bacteria based on protein family content
Source: Nat Commun. 2019 Sep 13;10:4173. doi: 10.1038/s41467-019-12171-z (PMC6744442; doi:10.1038/s41467-019-12171-z)
Supplement: Supplementary file 4 — Description of Additional Supplementary Files [file 41467_2019_12171_MOESM4_ESM.pdf]

Description of Additional Supplementary Files

File Name: Supplementary Data 1

Description: Annotation of the 921 widespread families. Column A: family accession. Column B: number of proteins in the family. Column C: median length of the proteins. Column D: ratio of proteins predicted to contain a signal peptide. Column E: median number of predicted transmembrane helix per protein. Column F: predicted cellular localization according to Psort. Column G: ggkbase annotation. Column H: domain architecture reported by Pfam. Columns I, J, K, L, M: KEGG annotations. Column N: TCDB annotation. Column O: Cazy annotation. Columns P,Q,R,S: number of CPR, non-CPR bacteria, DPANN, non-DPANN archaea genomes that carry the family in the initial dataset. Columns T, U, V and W: contingency table used for the enrichment analysis (Fisher test) using the initial dataset, adjusted p-value, odds-ratio and abundancy category of the family in the CPR relative to non-CPR bacteria (depleted, enriched or equally distributed). Columns X, Y, Z and AA: number of CPR, non-CPR bacteria, DPANN, non-DPANN archaea genomes that carry the family in the second "NCBI" dataset. Columns AB, AC, AD and AE: contingency table used for the enrichment analysis (Fisher test) using the second "NCBI" dataset, adjusted p-value, odds-ratio and abundancy category of the family in the CPR relative to non-CPR bacteria (depleted, enriched or equally distributed). Column AF: final and abundancy category of the family in the CPR relative to non-CPR bacteria (depleted, enriched or equally distributed).

File Name: Supplementary Data 2

Description: List of the prokaryotic genomes we used in the comparative analysis. For each genome, the taxonomy, accession and the levels of completeness and contamination are provided.
